# Direction and symmetry transition of the vector order parameter in topological superconductors Cu_*x*_Bi_2_Se_3_

**DOI:** 10.1038/s41467-019-14126-w

**Published:** 2020-01-13

**Authors:** T. Kawai, C. G. Wang, Y. Kandori, Y. Honoki, K. Matano, T. Kambe, Guo-qing Zheng

**Affiliations:** 1 Department of Physics, Okayama University, Okayama, 700-8530 Japan; 2 Institute of Physics, Chinese Academy of Sciences, and Beijing National Laboratory for Condensed Matter Physics, 100190 Beijing, China; 3 School of Physical Sciences, University of Chinese Academy of Sciences, 100190 Beijing, China

**Keywords:** Superconducting properties and materials, Topological insulators

## Abstract

Topological superconductors have attracted wide-spreading interests for the bright application perspectives to quantum computing. Cu_0.3_Bi_2_Se_3_ is a rare bulk topological superconductor with an odd-parity wave function, but the details of the vector order parameter **d** and its pinning mechanism are still unclear. Here, we succeed in growing Cu_*x*_Bi_2_Se_3_ single crystals with unprecedented high doping levels. For samples with *x*  = 0.28, 0.36 and 0.37 with similar carrier density as evidenced by the Knight shift, the in-plane upper critical field *H*_c2_ shows a two-fold symmetry. However, the angle at which the *H*_c2_ becomes minimal is different by 90° among them, which indicates that the **d**-vector direction is different for each crystal likely due to a different local environment. The carrier density for *x*  = 0.46 and 0.54 increases substantially compared to *x* ≤ 0.37. Surprisingly, the in-plane H_c2_ anisotropy disappears, indicating that the gap symmetry undergoes a transition from nematic to isotropic (possibly chiral) as carrier increases.

## Abstract (web summary)

The understanding of the vector order parameter for Cu_0.3_Bi_2_Se_3_ topological superconductor remains unclear. Here, Kawai et al. report a surprising disappearance of the in-plane upper critical field anisotropy for Cu_*x*_Bi_2_Se_3_ single crystals with more carriers, indicating a transition from nematic to chiral superconducting gap symmetry.

## Introduction

Exploring topological materials and their electronic functions are among the front-most topics of current condensed matter physics. In particular, much attention has been paid in recent years to topological superconductors where Majorana fermions (excitations) are expected to appear on edges or in the vortex cores^1,2^. Such novel edge states can potentially be applied to fault tolerant non-Abelian quantum computing^3,4^. So far, great success has been achieved in observing the Majorana bound state on the interface of a ferromagnet or a topological insulator in proximity to an *s*-wave superconductor^5–8^, or on the surface of iron-based superconductors^9^. In contrast, research on bulk topological superconductors progresses much more slowly. Candidates of bulk topological superconductors include superconductors with broken time reversal symmetry^10,11^, superconductors with broken spatial inversion symmetry^12,13^ and odd-parity superconductors with spatial inversion symmetry^14,15^. For the last case, the criteria for topological superconductivity are effectively two fold. Namely, odd-parity of the gap function and an odd number of time-reversal invariant momenta in the Brillouin zone^14^. Experimentally, clear evidence for odd-parity superconductivity had not been found until very recently^16^. Although Cu-doped topological insulator Cu_*x*_Bi_2_Se_3_^17^ had been proposed as a candidate^14^, experiments had been controversial^18–20^.

The discovery of spontaneous spin rotation-symmetry breaking in the bulk superconducting state of Cu_0.3_Bi_2_Se_3_ by nuclear magnetic resonance (NMR) measurements established the spin-triplet, odd-parity superconducting state^16^. Since there is only one time-reversal-symmetric momentum in the Brillouin zone of Cu_*x*_Bi_2_Se_3_^21^, this material fulfills the two-fold criteria and can be classified as a topological superconductor. However, detailed gap function is still unclear. If the gap is fully-opened, then Cu_0.3_Bi_2_Se_3_ is a class DIII topological superconductor^22^, where Majorana zero-energy modes are expected at edges or vortex cores. If there are nodes in the gap function, the material is nonetheless topological just as the cases of Dirac or Weyl semimetals.

A more generally-used term associated with the gap in a spin-triplet superconductor is the vector order parameter **d**, whose direction is perpendicular to the direction of paired spins and whose magnitude is the gap size. The **d**-vector was found to be parallel to *a*-axis (the Se–Se bond direction) in Cu_0.3_Bi_2_Se_3_^16^. This was the first case where the **d**-vector direction was unambiguously determined in any spin-triplet superconductor candidate. The emergent two-fold symmetry in the Knight shift below *T*_c_^16^ was interpreted by the concept of nematic order^23^, and had triggered many subsequent extensive studies on rotational symmetry breaking by various methods, which also revealed a two-fold symmetry in other physical properties^24–28^. Measurements by transport^29^, penetration depth^30^, and scanning tunneling microscope (STM)^31,32^ suggesting unconventional superconductivity have also been reported since then.

However, why the **d**-vector is oriented to one of *a*-axes, and why it is robust against heat cycle through the superconducting transition, remain unknown. Note that there are three equivalent *a*-axis directions. This issue is important as the gap symmetry (“nematicity” indicator) is closely tied to the direction of the **d**-vector^23^. From material point of view, it had been unclear whether the carrier density can be controlled and how the physical properties would change with changing carrier density. A previous report showed that the Hall coefficient does not change even though the nominal *x* increases from 0.15 to 0.45^33^. These are the issues we wish to address in this article.

In this work, we synthesized Cu-doped Bi_2_Se_3_ single crystals by the electrochemical intercalating method. Through the measurements of the Knight shift, we find unprecedentedly that the carrier concentration further increases with increasing *x* beyond *x* = 0.37. We study the angle dependence of the upper critical field *H*_c2_ in different crystals. For an odd-parity gap function, the gap anisotropy will lead to an anisotropic *H*_c2_. One then can obtain knowledge about how the superconducting gap evolves with *x* by measuring the *H*_c2_ anisotropy. For samples with *x* = 0.28, 0.36, and 0.37 which have the same size of the Knight shift, we find a two-fold symmetry in the in-plane *H*_c2_ by ac susceptibility and magnetoresistance measurements, in agreement with previous reports^16,24–28^. However, the angle at which *H*_c2_ is a minimum differs by 90°, which means that the direction of the **d**-vector is different for each crystal. In contrast, for *x* = 0.46 and 0.54, two-fold anisotropy disappears, which indicates a nematic-to-isotropic transition of the gap symmetry as carrier density increases. We discuss possible exotic (chiral) superconducting state for the samples with large *x*.

## Results

### Sample characterization

**Fig. 1:**
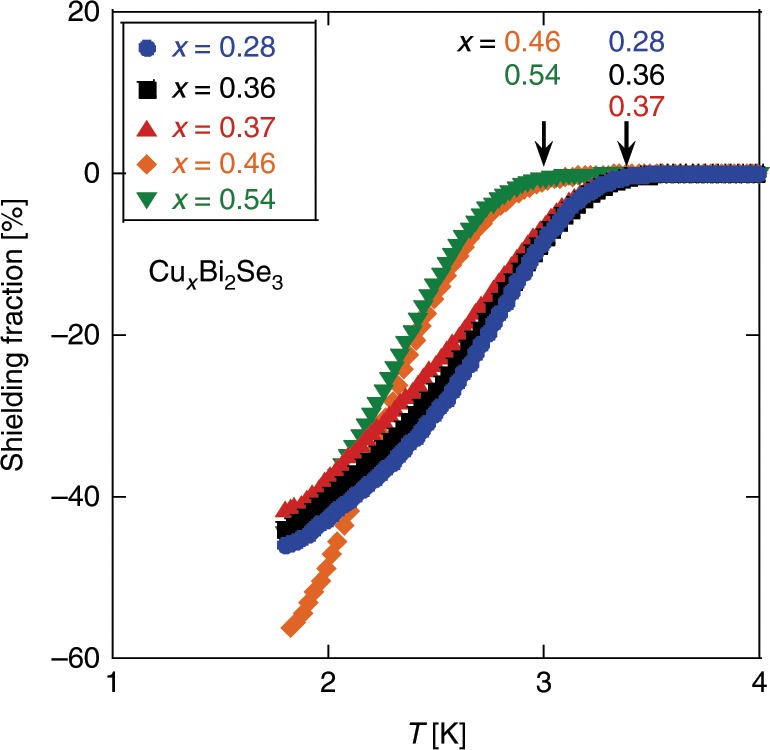
Diamagnetism measurements. Shielding fraction for five samples with different *x*. The arrows indicate *T*_c_.

**Fig. 2:**
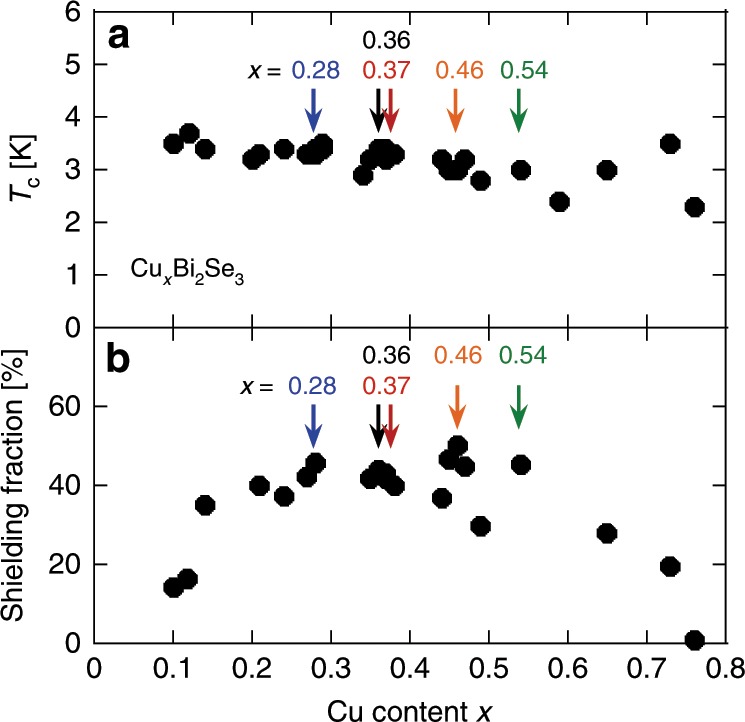
Properties of the samples. Cu content (*x*) dependence of (**a**) the superconducting critical temperature *T*_c_ and (**b**) the shielding fraction at 1.8 K. The arrows indicate samples used for the *H*_c2_ and NMR measurements reported in this paper.

Figure 1 shows the result of dc susceptibility measurements for representative samples. In Fig. 2, we summarize the properties of all the samples we synthesized. The obtained *T*_c_ and shielding fraction (SF) for most samples are close to the values reported by Kriener et al.^34^. The SF for *x* = 0.46 is the highest (56.2%) among those reported so far. In Table 1 we list the properties for the five samples that we will discuss in this paper.

**Table 1:** The *T*_c_ and the shielding fraction at *T* = 1.8 K for the five Cu contents discussed in this paper.

Cu content *x*	0.28	0.36	0.37	0.46	0.54
*T*_c_ [K]	3.4	3.4	3.4	3.0	3.0
Shielding fraction [%]	45.9	44.1	41.5	56.2	44.7

**Fig. 3:**
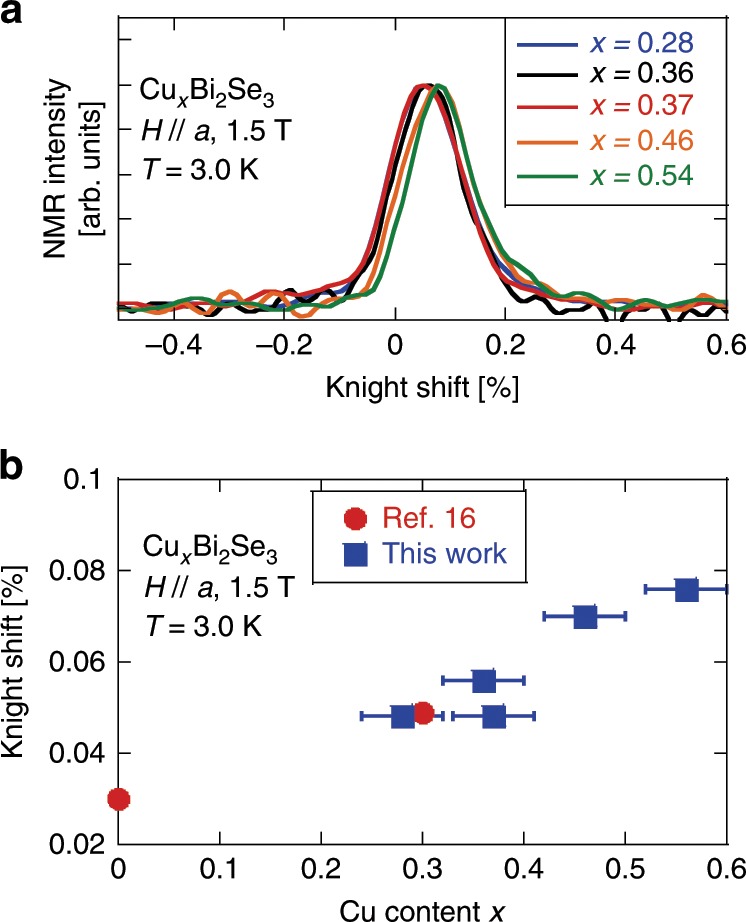
NMR Spectra and Knight shift. **a** The ^77^Se-NMR spectra measured in a magnetic field of *H*_0_ = 1.5 T. **b** Cu content *x* dependence of the Knight shift. Data for *x* = 0 and 0.3 are from ref. ^16^. The error for Cu-content *x* was determined by the resolution of the electronic balance used for measuring the weight of samples.

Figure 3a shows the ^77^Se(*I* = 1∕2)-NMR spectra above *T*_c_ for five samples with different *x*. Each spectrum can be fitted by a single Gaussian. The Knight shift is  ~0.048% for *x* = 0.28, 0.36, and 0.37, which is close to the value (*K* = 0.049%) previously reported for the sample with *x* = 0.3^16^. On the other hand, the Knight shift increased substantially in the samples with *x* = 0.46 and 0.54. Generally, the Knight shift is expressed as,

1 $$K = {K}_{{\rm{orb}}}+{K}_{{\rm{s}}},$$

2 $${K}_{{\rm{s}}}= {A}_{{\rm{hf}}}{\chi }_{{\rm{s}}},$$

where *K*_orb_ is the contribution due to orbital susceptibility and *A*_hf_ is the hyperfine coupling constant, which are independent of carrier density. The *χ*_s_ is the spin susceptibility which is proportional to electronic density of states. For this field configuration, *K*_orb_ = 0.03%^16^. In Fig. 3b, we plotted the Knight shift as a function of Cu content *x*. Upon doping, *K* increases as compared to the parent compound, which implies that the carrier is indeed doped into the sample. The fact that *K* has a similar value for the samples with *x* = 0.28–0.37 indicates that the carrier density does not change appreciably in this *x* region. Such behavior is consistent with other reports by different methods^33,35^. On the other hand, in the high-*x* region, we discovered for the first time that carriers increase further as *x* increases beyond 0.37.

We briefly comment on possible mechanisms of Cu-doping. In Sr-doped system^35^, Sr goes into multiple sites, namely, the intercalated site in-between the quintuple layer blocks, interstitial site, or the in-plane site substituting for Bi. Cu(Sr) residing on the intercalated or interstitial site contributes electrons, while Cu(Sr) going to the substituting site contributes holes^17^. The stage-like *x*-dependence of *K* suggests that Cu doping mechanism in each *x*-range is not simple, but rather Cu may also go into multiple sites. As post annealing is necessary after electrochemical process in the present case, we speculate that Cu may migrate into a different site after annealing, resulting in the peculiar behavior of *K* with respect to *x*. The Cu position is one of the issues that needs to be addressed in future works.

### Anisotropy of the upper critical field *H*_c2_ and its disappearance

**Fig. 4:**
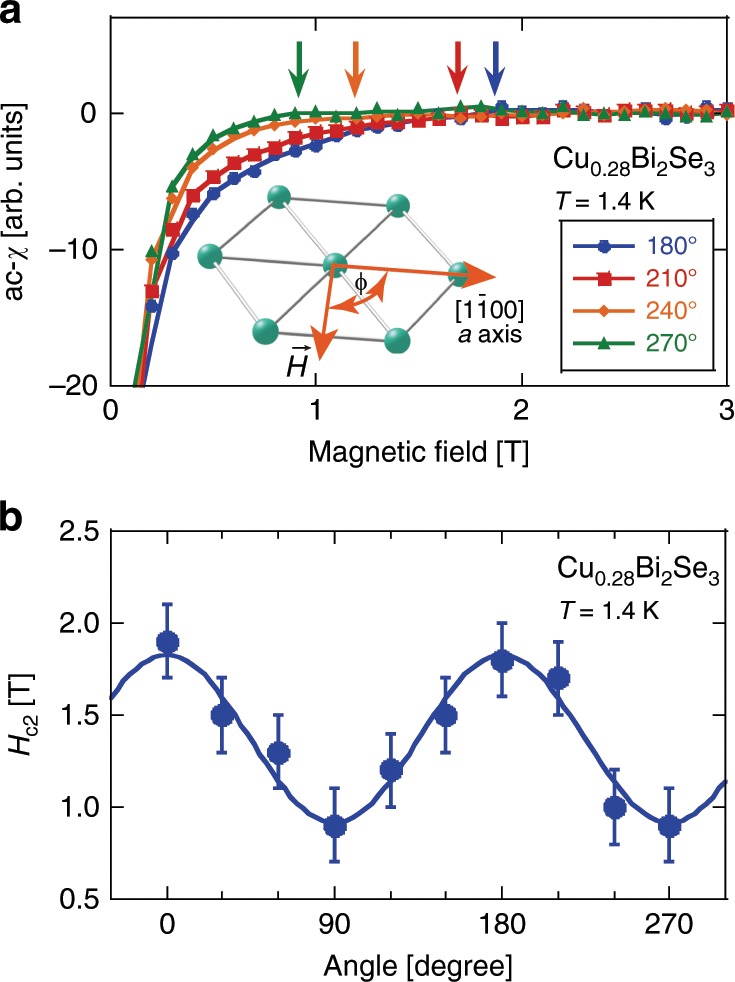
In-plane upper critical field anisotropy determined by magnetic susceptibility. **a** Magnetic field dependence of the ac-*χ* at different in-plane angle *ϕ* for the sample with *x* = 0.28. The solid curves are guides to the eyes. The arrows indicate *H*_c2_ for each angle, which is defined as a point off the straight line drawn from high-field value (the normal state). The error was estimated by assuming that the uncertainty equals twice point-interval in measuring the ac-*χ* *vs* field curve. For more details see Supplementary Information. The inset is an illustration depicting the hexagonal plane and the angle *ϕ*. **b** *H*_c2_ plotted as a function of in-plane angle *ϕ*. The blue curve is a sine function.

Next we present data on the anisotropy of *H*_c2_. For *x* = 0.28, we measured both ac susceptibility (ac-*χ*) and electrical resistance by changing the magnetic field for each field-direction relative to *a*-axis. The inset to Fig. 4a shows the angle *ϕ* between the applied magnetic field *H* and the *a*-axis. Before Cu doping, the direction of *a*-axis (Se–Se bond direction) was determined by Laue diffraction, which we assign as *ϕ* = 0°. The main panel of Fig. 4a shows the magnetic field dependence of the ac-*χ* at different *ϕ* for *x* = 0.28. As can be seen in the figure, the field dependence of ac-*χ* is clearly different for each angle below a certain field (*H*_c2_), while above *H*_c2_ the *H*-dependence of ac-*χ* is the same for all angles. Fig. 4b shows *H*_c2_ as a function of angle. A clear two-fold symmetry is observed.

**Fig. 5:**
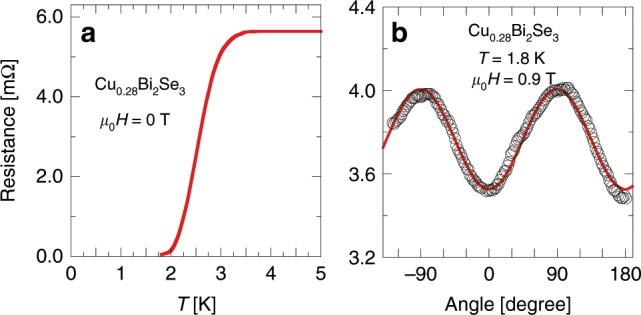
Magneto-resistance measurements. **a** Temperature dependence of the electrical resistance at zero magnetic field, which is in agreement with ref. ^34^. **b** Electrical resistance under a magnetic field of 0.9 T and at *T* = 1.8 K as a function of in-plane angle *ϕ*. Note that a minimum in the electrical resistance corresponds to a maximal *H*_c2_. The red curve is a sine function.

**Fig. 6:**
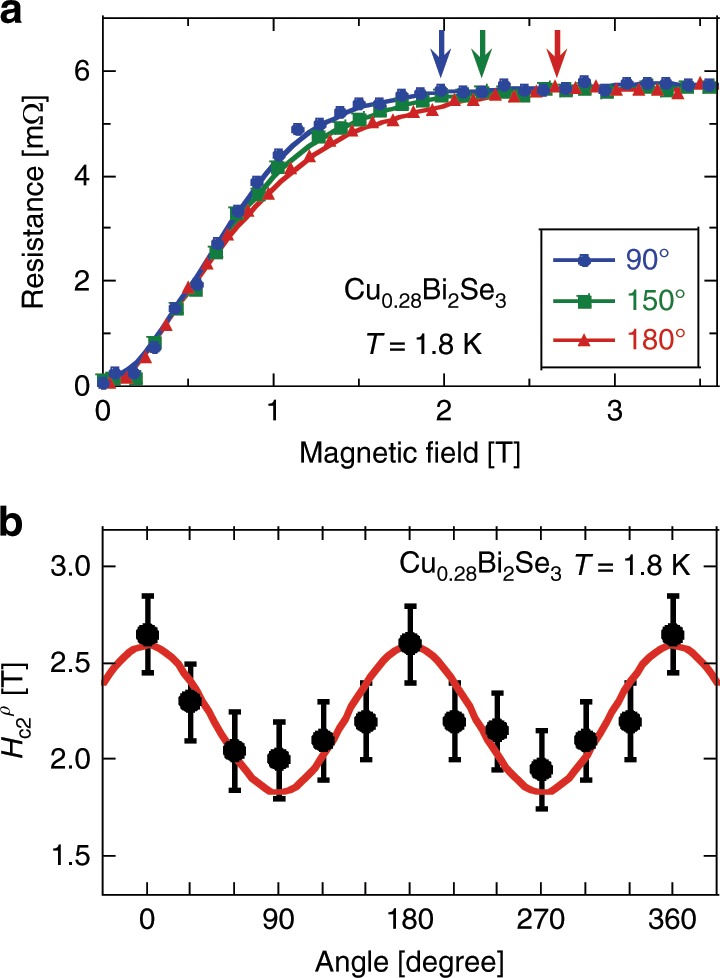
In-plane upper critical field anisotropy determined by magneto-resistance. **a** Electrical resistance at *T* = 1.8 K as a function of the magnetic field for three representative in-plane angles. The arrows indicate *H*_c2_  which is defined as the field below which the data point deviates from a straight line drawn from the normal state. **b** Upper critical field ${H}_{{\rm{c}}2}^{\rho }$ as defined in (**a**) is plotted as a function of in-plane angle. The error bar was estimated by assuming that the uncertainty equals twice point-interval in measuring the resistance vs. field curve. The red curve is a sine function.

Such two-fold symmetry is also seen in the magnetoresistance measurements, as demonstrated in Fig. 5 where the electrical resistance under a field of 0.9 T and at *T* = 1.8 K is plotted as a function of angle. We checked the angle-resolved resistance at *μ*_0_*H* = 3 T and *T* = 1.8 K where the sample is in the normal state and found only a random noise with an amplitude no bigger than 0.1 mΩ, thus confirming that such two-fold oscillation in the resistance shown in Fig. 5b is caused by the anisotropy of *H*_c2_. Fig. 6a shows the resistance as a function of magnetic field at *T* = 1.8 K for three representative angles. Similar to the results shown in Fig. 4a, a clear angle dependence is found. Fig. 6b shows the angle dependence of ${H}_{{\rm{c}}2}^{\rho }$ obtained from the magnetoresistance data. A two-fold symmetry is also clearly seen. This result is in agreement with that seen in the ac-*χ* data shown in Fig. 4b, although such-defined ${H}_{{\rm{c}}2}^{\rho }$ is slightly higher but is not surprising for its definition. An in-plane anisotropy of *H*_c2_ is also resolved from the data of electrical resistance *vs* temperature under different magnetic fields (see Supplementary Figs. 1, 2).

**Fig. 7:**
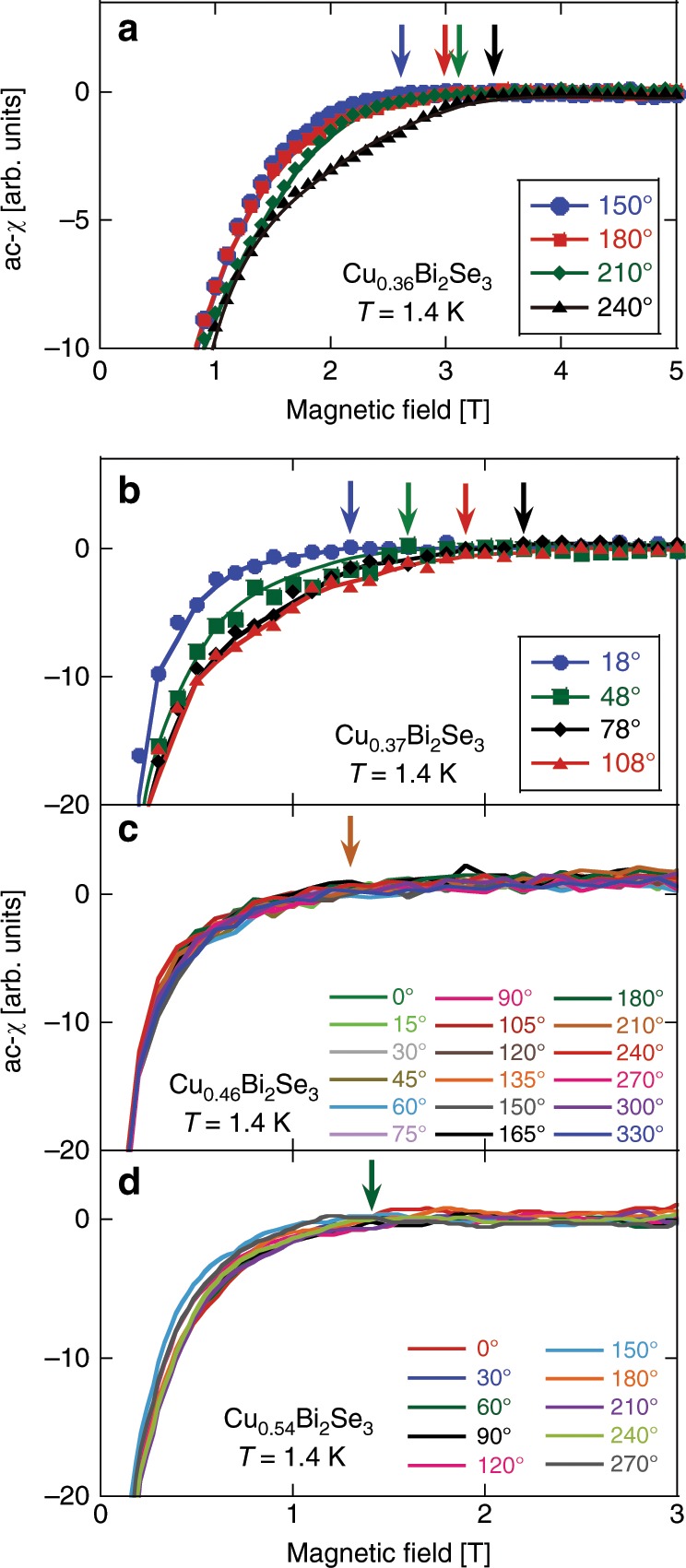
Magnetic field dependence of the ac-*χ* at different angle *ϕ* for *x* = 0.36, 0.37, 0.46, and 0.54. The solid curves are guides to the eyes. For sake of clarity, the data points for *x* = 0.46 and 0.54 are represented by symbols with minimized size. The arrows indicate *H*_c2_ defined in the same way as previous figures. For *x* = 0.46 and 0.54, the arrow is for *ϕ* = 15 and 90°, respectively.

Quite often, extracting *H*_c2_ from the magnetic susceptibility has several advantages over that from magnetoresistance measurements. Firstly, the magnetic susceptibility is more sensitive to superconducting volume fraction rather than surface. Secondly, in two-dimensional or layered superconductors, *H*_c2_ determined by resistivity measurements is often inaccurate because of vortex lattice melting^36^. For example, in high-*T*_c_ cuprates, even in the resistive state, one is still in a regime dominated by Cooper pairings in the presence of vortices^36^. For these reasons and the technical merit that ac-*χ* can be measured to a lower temperature in our case, below we discuss the evolution of gap symmetry based on the ac-*χ* data. Data for *x* = 0.36, 0.37, 0.46, and 0.54 are shown in Fig. 7. For *x* = 0.36 and 0.37, clear angle dependence was found as in *x* = 0.28. In contrast, the tendency is completely different for samples with larger *x*. There is no angle dependence in ac-*χ* for the samples with *x* = 0.46 and 0.54.

**Fig. 8:**
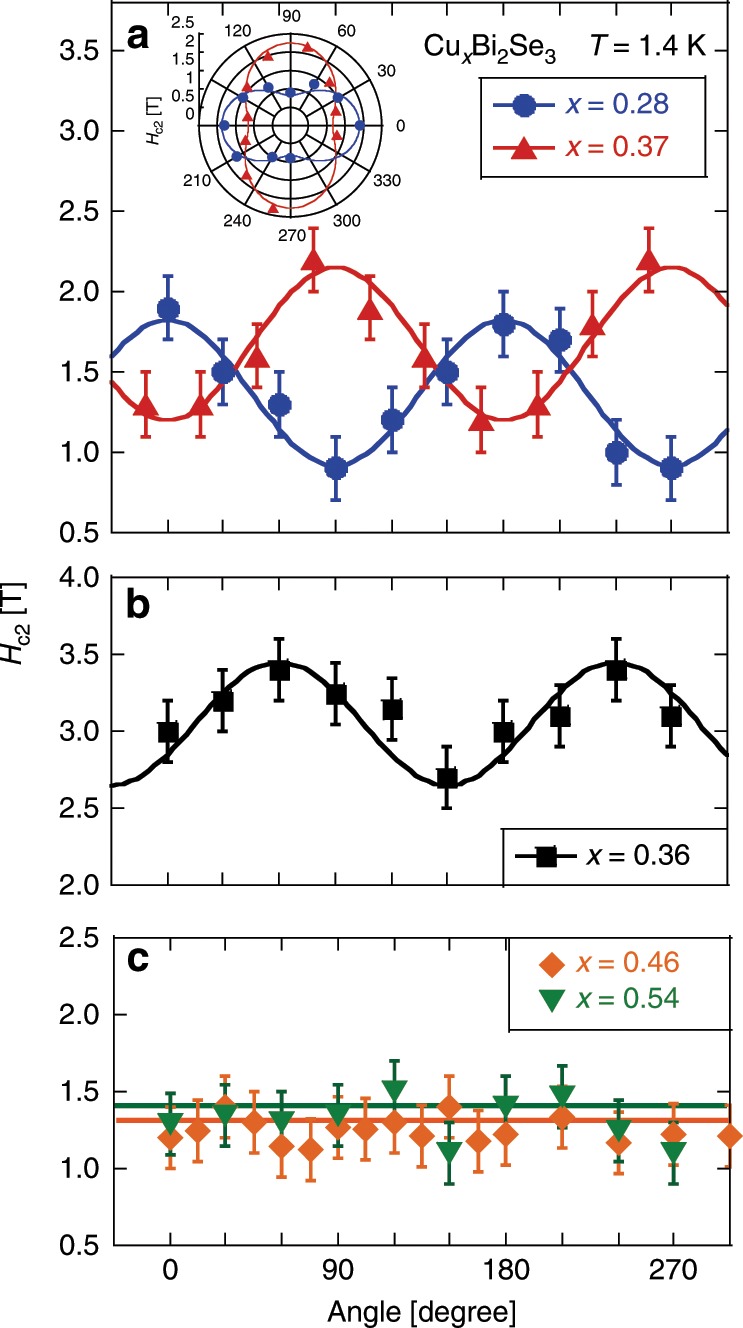
In-plane *H*_c2_ at *T* = 1.4 K extracted from ac-*χ* as a function of angle *ϕ* for all samples. The error bar was estimated by assuming that the uncertainty equals twice point-interval in measuring the ac-*χ vs* field curve. The solid curves are sine functions. The inset to (**a**) is a polar plot of the angular dependence *H*_c2_ for *x* = 0.28 and 0.37.

The angular dependence of *H*_c2_ at 1.4 K determined from the ac-*χ* for all samples is plotted in Fig. 8 (for more details, see Supplementary Fig. 3). For the samples with smaller *x* (Fig. 8a, b), *H*_c2_ shows a large and two-fold anisotropy. In contrast, for larger *x* (Fig. 8c), no anisotropy is observed. The oscillation amplitude of *H*_c2_ is similar for *x* = 0.28, 0.36, and 0.37. The magnitude of *H*_c2_ is also similar between *x* = 0.28 and 0.37, but is larger for *x* = 0.36. The origin of this difference is unknown at the moment.

Interestingly, the angle at which *H*_c2_ becomes minimal is 90° and 150° (perpendicular to Se–Se bond) for *x* = 0.28 and 0.36, respectively, but is 0° (along Se–Se bond) for *x* = 0.37. For this crystal structure, the equivalent crystal-axis direction appears every 60°. If two oscillation patterns have a phase difference of 60°, it can be said that they are the same crystallographically. Therefore, the samples with *x* = 0.28 and 0.36 have the same gap symmetry. However, 90° difference means that the gap symmetry is different. It is noted that the angle at which *H*_c2_ is minimal corresponds to the direction of **d**-vector^16^. Therefore, our result indicates that the **d**-vector direction differs for each sample in Cu_*x*_Bi_2_Se_3_ even though the carrier density is the same or very similar. The *x* = 0.37 sample has the same symmetry as the previous sample used for NMR measurement where the **d**-vector is pinned to Se–Se direction^16^, while the *x* = 0.28 and 0.36 samples have the same symmetry as the sample used for specific heat measurements^24^.

### No multiple domains

**Fig. 9:**
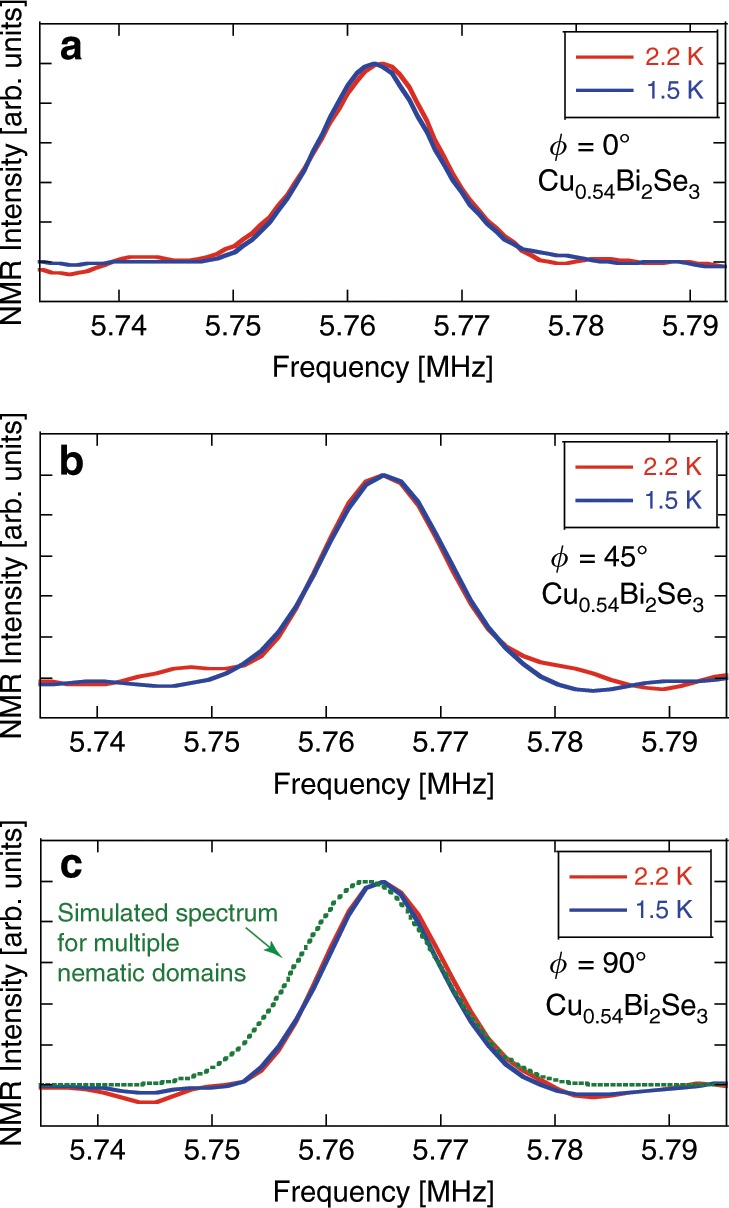
NMR spectra and simulation. NMR spectra at *T*_c_ (*H* = 0.7 T) = 2.2 K and below for *x* = 0.54 with different angles between *H* and the *a*-axis (**a**–**c**). **c** A simulation for the spectrum in presence of multiple nematic domains, simply assuming that various domains are distributed randomly.

As for the disappearance of oscillation in *H*_c2_ for *x* = 0.46 and 0.54, a possibility of multiple domains each of which is a nematic phase with **d**-vector pointing to a different direction, can be ruled out. In that case, the NMR spectrum for *T* < *T*_c_ would be broadened compared to that at *T* > *T*_c_. This is because the spectrum coming from the domains with *H*∥ **d**-vector will shift to a lower frequency^16^, but that from domains with *H* ⊥ **d**-vector will not. However, our result (Fig. 9a–c) shows no such broadening which is not compatible with the multiple-domains scenario. In Fig. 9c, we show a simulation for the spectrum in case of multiple domains, assuming that various domains are distributed randomly as to wipe out oscillations in the angle dependence of *H*_c2_. The simulation also assumed that, for *H*∥ **d**-vector, the spin Knight shift (*K*_s_) below *T*_c_ follows the same temperature dependence as found in Cu_0.3_Bi_2_Se_3_^16^; *K*_s_ = 0.046% and the reduction of *K*_s_ at *T* = 1.5K, *ΔK*_s_ = 0.043%, were used in the simulation.

## Discussion

Fu pointed out that the spin rotation-symmetry breaking (the in-plane Knight shift nematicity) can be understood if the pairing function is a doublet representation^23^, *Δ*_4*x*_ or *Δ*_4*y*_, both of which being *p*-wave. For *Δ*_4*x*_ state, the **d**-vector is along the principle crystal axis while it is orthogonal to the principle crystal axis for *Δ*_4*y*_ state. In the *Δ*_4*x*_ state, there are two point nodes in the superconducting gap, which are in the direction perpendicular to the Se–Se bond. In the *Δ*_4*y*_ state, there is no node but a minimum in the superconducting gap, and the **d**-vector is oriented perpendicular to the gap-minimum direction. Applying Fu’s theory to our results, the *x* = 0.37 and the previous *x* = 0.3^16^ samples would correspond to *Δ*_4*x*_, and the samples with *x* = 0.28 and 0.36 would correspond to *Δ*_4*y*_ state.

As mentioned in ref. ^23^, the theory does not take crystalline anisotropy into consideration so that *Δ*_4*x*_ and *Δ*_4*y*_ are degenerate. In real crystals, however, crystalline distortions due to dopants exist, and it was suggested that the dopant position is important for superconductivity^35,37^. Hexagonal distortion was indeed reported in Sr_*x*_Bi_2_Se_3_^38^. The same can be expected in Cu_*x*_Bi_2_Se_3_^39^. Moreover, strains induced by quenching can vary from one sample to another. The chemical processes to obtain a sample with high SF are complex which includes a quenching process. Only those quenched from a narrow temperature (~560 °C) show high SF, which suggests that it is important to seize a metallurgically meta-stable phase to obtain the superconductivity. However, the quenching process is less controllable so that strain induced during this process is random among samples. We believe that **d**-vector pointing to different directions in samples with the same carrier density is due to a different local structural environment such as strain caused by quenching, dopant-induced crystal distortion, etc.

The most intriguing and surprising finding of this work is that the oscillation in *H*_c2_ disappears for the samples with *x* = 0.46 and 0.54, as seen in Fig. 8b. Judging from the NMR spectrum width which is almost the same for *x* = 0.36, 0.48, and 0.54 in particular, we conclude that the sample homogeneity is very similar among them. Also, a multiple-domains scenario can be ruled out as discussed in the previous section. We interpret the surprising evolution of *H*_c2_ as due to an emergent fully-opened isotropic gap for large *x*. It was previously pointed out that the odd-parity superconductivity with two-component *E*_u_ representation admits two possible phases, nematic and chiral^40,41^. Under some circumstances, a chiral, fully-gapped, state becomes more stable as compared to a nematic state. One of the crucial parameters important for selecting between the two states is the Fermi surface shape. It has been theoretically shown by several groups that the chiral state can be stabilized when the Fermi surface becomes more two-dimensional^42–44^. Experimentally, photoemission and quantum oscillation measurements have suggested that the Fermi surface becomes more two-dimensional as carrier density increases^45,46^. Indeed, the Knight shift result indicates that carriers increased in the samples of *x* = 0.46 and 0.54 as compared to *x* ≤ 0.37. Therefore, the dramatic change in the angle dependence of the *H*_c2_ can be naturally explained as due to a gap symmetry change from nematic to chiral. The lack of change of the Knight shift below *T*_c_ for all angles (Fig. 9) as contrary to what found in the nematic phase^16^ is consistent with existing theory that the **d**-vector for the chiral phase would be pointing to the *c*-axis^42^. Thus, our finding suggests that superconductors with strong spin-orbital interaction^14^ is a land much more fertile than we thought.

In summary, single crystal samples of Cu_*x*_Bi_2_Se_3_ with unprecedented high doping levels were newly synthesized and investigated. By NMR measurements, we found for the first time that the carrier density increased further by Cu doping beyond *x* = 0.37. By magnetic susceptibility measurements, we found that the in-plane *H*_c2_ shows a clear two-fold oscillation for the samples with *x* = 0.28, 0.36, and 0.37 which have similar carrier density as evidenced by the Knight shift. However, the angle at which *H*_c2_ becomes minimal is different by 90° between different samples. This indicates that the direction of the **d**-vector is different from crystal to crystal due to a different local structure caused by strain during the quenching process, dopant-induced crystal distortion, etc. In the samples with *x* = 0.46 and 0.54, the two-fold oscillation is completely suppressed, which indicates a gap symmetry change from nematic to isotropic as carrier density increases. These findings enriched the contents of topological superconductivity in doped Bi_2_Se_3_, and we hope that our work will stimulate further studies on possibly even more exotic superconducting state (possible chiral state) in the high-doped region of Cu_*x*_Bi_2_Se_3_ as well as on bulk topological superconductors in general.

## Methods

### Single crystal growth and characterization

Single crystals of Cu_*x*_Bi_2_Se_3_ were prepared by intercalating Cu into Bi_2_Se_3_ by the electrochemical doping method described in ref. ^34^. First, single crystals of Bi_2_Se_3_ were grown by melting stoichiometric mixtures of elemental Bi (99.9999%) and Se (99.999%) at 850 °C for 48 h in sealed evacuated quartz tubes. After melting, the sample was slowly cooled down to 550 °C over 48 h and kept at the same temperature for 24 h. Those melt-grown Bi_2_Se_3_ single crystals were cleaved into smaller rectangular pieces of about 14 mg. They were wound by bare copper wire (dia. 0.05 mm), and used as a working electrode. A Cu wire with diameter of 0.5 mm was used both as the counter (CE) and the reference electrode (RE). We applied a current of 10 μA in a saturated solution of CuI powder (99.99%) in acetonitrile (CH_3_CN). The obtained crystals samples were then annealed at 560 °C for 1 h in sealed evacuated quartz tubes, and quenched into water. After quenching, the samples were covered with epoxy (STYCAST 1266) to avoid deterioration. The Cu concentration *x* was determined from the mass increment of the samples. To check the superconducting properties, dc susceptibility measurements were performed using a superconducting quantum interference device (SQUID) with the vibrating sample magnetometer (VSM).

### NMR measurements

The ^77^Se-NMR spectra were obtained by the fast Fourier transformation of the spin-echo at a fixed magnetic field (1.5 T or 0.7 T). The Knight shift *K* was calculated using nuclear gyrometric ratio *γ*_N_  = 8.118 MHz/T for ^77^Se.

### Angle-resolved *H*_c2_ measurements

*H*_c2_ was determined from ac susceptibility by measuring the inductance of in-situ NMR coil. Angle-dependent measurements were performed by using a piezo-driven rotator (Attocube ANR51) equipped with Hall sensors to determine the angle between magnetic field and crystal axis. The ac-*χ* vs. *H* data in the normal state were fitted by a linear function (a constant line). *H*_c2_ was defined as a point off the straight line. A typical example is shown in Supplementary Fig. 3.

### Magnetoresistance measurements

The angle-dependent electrical resistance was measured by the standard four-electrode method in a physical properties measurement system (PPMS, Quantum Design) with a mechanical rotating probe. The building of electrodes were carried out in a glove box filled with high-purity Ar gas to prevent sample from degradation. The electrodes were made such that the current direction is along the *a*-axis. The excitation currents are 0.1–1 mA to make a compromise of the Joule heating and the measurement accuracy.

## Supplementary information

Supplementary Information

## Data Availability

The data that support the findings of this study are available on reasonable request.
