# Supplementary material for: Direction and symmetry transition of the vector order parameter in topological superconductors CuxBi2Se3
Source: Nat Commun. 2020 Jan 13;11:235. doi: 10.1038/s41467-019-14126-w (PMC6957487; doi:10.1038/s41467-019-14126-w)
Supplement: Supplementary file 1 — Supplementary Information [file 41467_2019_14126_MOESM1_ESM.pdf]

Direction and symmetry transition of the vector order parameter in topological superconductors $Cu_xBi_2Se_3$

T. Kawai *et al*

SUPPLEMENTARY INFORMATION

Direction and symmetry transition of the vector order parameter in topological superconductors $Cu_xBi_2Se_3$

T. Kawai [1], C. G. Wang [2,3], Y. Kandori [1], Y. Honoki [1],K. Matano [1], T. Kambe [1], and Guo-qing Zheng [1,2]

[1] *Department of Physics, Okayama University, Okayama 700-8530, Japan*
[2] *Institute of Physics, Chinese Academy of Sciences, and Beijing National Laboratory for Condensed Matter Physics, Beijing 100190, China*
[3] *School of Physical Sciences, University of Chinese Academy of Sciences, Beijing 100190, China*

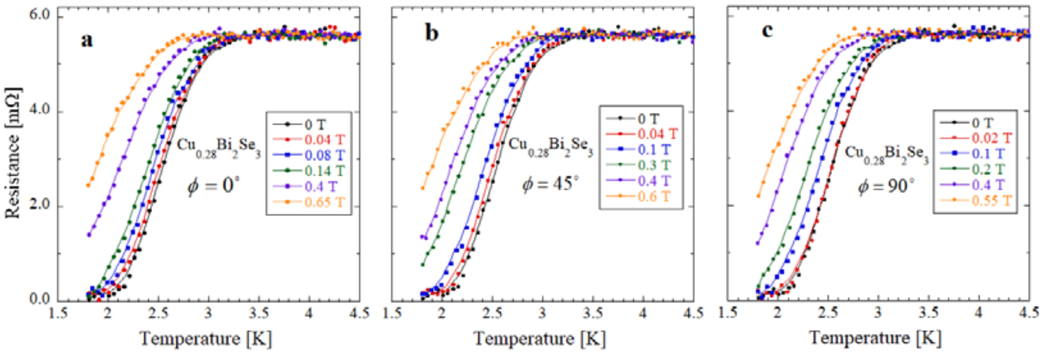

Supplementary Figure 1. Electrical Resistance under various magnetic fields applied along different directions with respect to the a-axis. The figures show the temperature dependence of the electrical resistance with the magnetic field applied at an angle of 0, 45 and 90 degrees with respect to the *a*-axis.

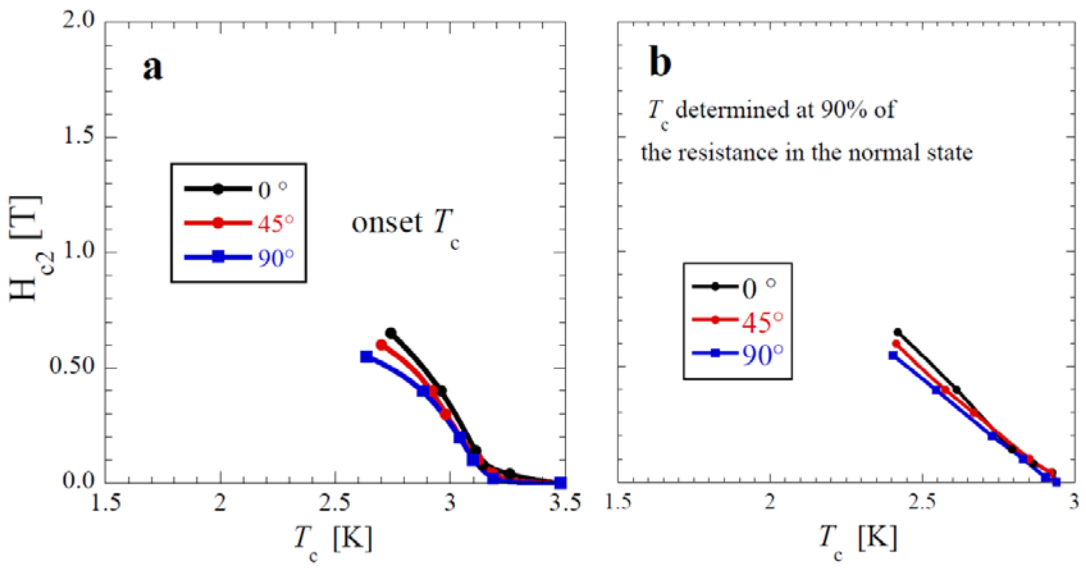

Supplementary Figure 2. Upper critical field extracted from Fig. S1. H_{c2} as a function of T_c for the angles of 0, 45 and 90 degrees with respect to the *a*-axis. For (a), T_c is defined as a point off the line drawn from the normal state value.

For (b), T_c is defined as a point where the resistance drops to 90% of the value at T=5 K in the normal state. In both cases, an in-plane anisotropy of H_{c2} is resolved.

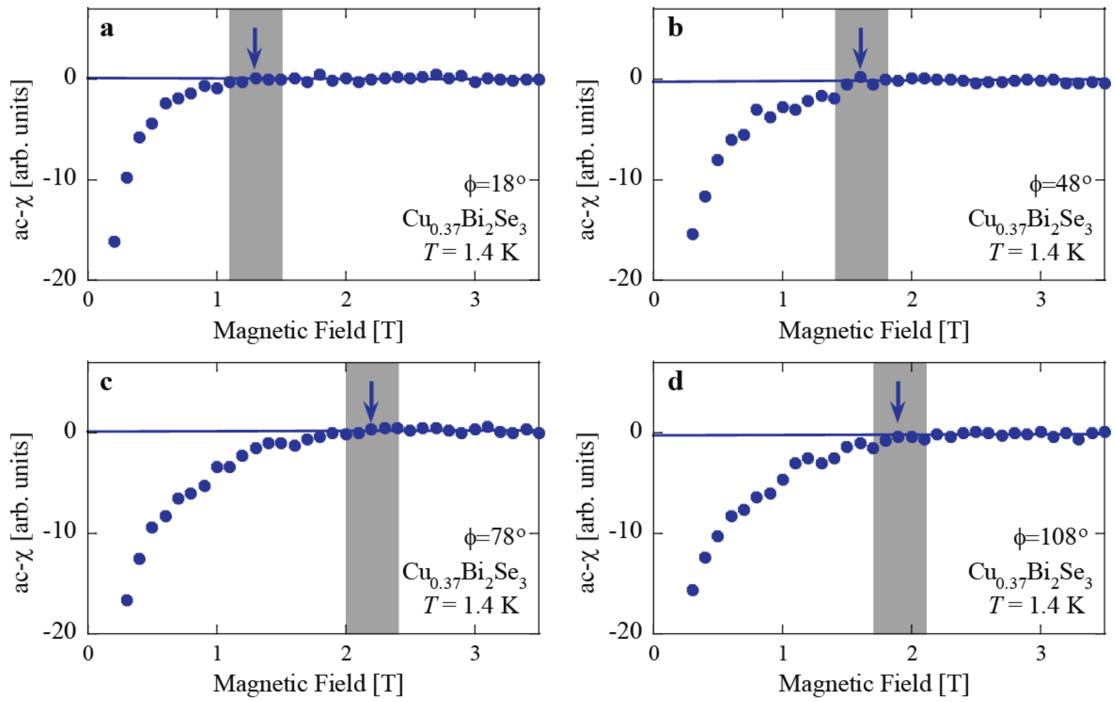

Supplementary Figure 3. Examples of the determination of H_{c2}. Magnetic field dependence of the ac-χ at different angle ϕ for x =0.37. The solid lines are linear fitting of the data in the normal state. The arrows indicate H_{c2} for each angle, which is defined as a point off the straight line. Shaded area represents error bar in estimating H_{c2}.
